# Systemic Lupus Erythematosus and Psoriasis Overlap: A Case Series

**DOI:** 10.7759/cureus.84760

**Published:** 2025-05-24

**Authors:** Mohannad Ali, Saud Abdullah, Yasameen Abdulhameed, Faisal Elbadawi

**Affiliations:** 1 Internal Medicine, DubaiHealth, Dubai, ARE; 2 Rheumatology, DubaiHealth, Dubai, ARE

**Keywords:** biologics, immunology, overlap, psoriasis, systemic lupus erythematosus

## Abstract

Systemic lupus erythematosus (SLE) is a chronic multisystem autoimmune disorder known for its diverse clinical manifestations and potential for overlap with other connective tissue diseases. While overlap syndromes involving conditions such as rheumatoid arthritis (RA) and Sjögren’s syndrome (SS) are well established, the coexistence of SLE and psoriasis remains relatively rare and under-reported, despite their distinct immunopathological mechanisms.

We present a case series of four female patients diagnosed with both SLE and psoriasis based on expert medical opinion along with supporting biochemical evidence, who experienced complex, relapsing-remitting disease courses. Each case demonstrated unique challenges related to diagnosis, overlapping cutaneous and joint symptoms, and therapeutic management. Overall, we have noted that the use of biological therapy in our patients was effective in terms of clinical remission of psoriasis while avoiding the risk of an SLE flare.

## Introduction

Systemic lupus erythematosus (SLE) is a complex chronic autoimmune disorder characterized by widespread inflammation and immune mediated injury to multiple organ systems secondary to dysregulated immune responses. It has a complex multifactorial pathogenesis that includes a combination of genetic, environmental, and hormonal factors that lead to development of autoimmunity [1].

The clinical presentation can widely vary from mild symptoms such as fatigue, skin manifestations and poly articular arthritis to severe life-threatening lupus nephritis and serositis [2]. It is well established that SLE can coexist with other connective tissue diseases in an entity called overlap syndromes. The most frequently overlap syndromes with SLE include rheumatoid arthritis (RA), Sjögren’s syndrome (SS), systemic sclerosis (SSc), dermatomyositis (DM), and polymyositis (PM) [3]. The overlap between SLE and psoriasis is relatively rare and is of particular interest considering the distinct pathophysiological mechanisms of the two diseases. We present a case series of four patients with SLE and psoriasis overlap who presented to our center.

## Case presentation

Case 1 

A 30-year-old female patient was diagnosed with SLE in 2012 based on joint pain and positive serology including antinuclear antibody (ANA), anti-double stranded DNA (anti-dsDNA) antibodies, and low complement levels. Initial treatment included hydroxychloroquine and steroids. Over the years, her disease course was complicated by lupus nephritis (Stage IIIA and Stage V) and avascular necrosis of the shoulders and hips. She received multiple immunosuppressants, including mycophenolate mofetil, rituximab, belimumab, cyclosporine, and cyclophosphamide.

**Figure 1:**
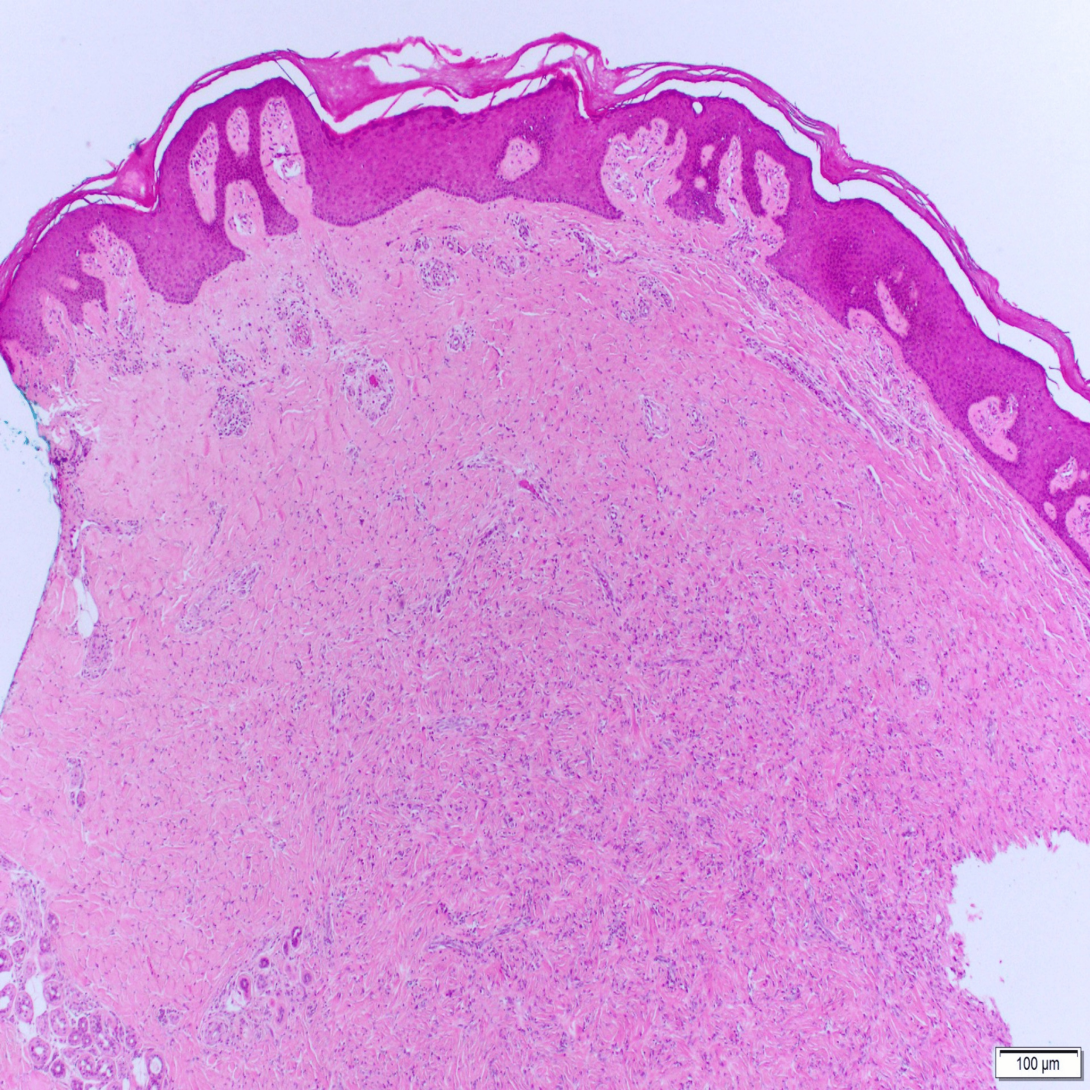
Skin biopsy showing features of psoriasiform dermatitis Microscopic description reveals regular acanthosis with elongated rete (psoriasiform). There is alternating zones of hypo and hypergranulosis in the epidermis. Areas of parakeratosis in the stratum corneum with mounds of neutrophils. There is mild perivascular lymphocytic infiltrate in the upper portion of the dermis. Hair follicles are seen surrounded by mild chronic inflammation with areas of derma fibrosis. The subcutaneous fat is unremarkable.

**Figure 2:**
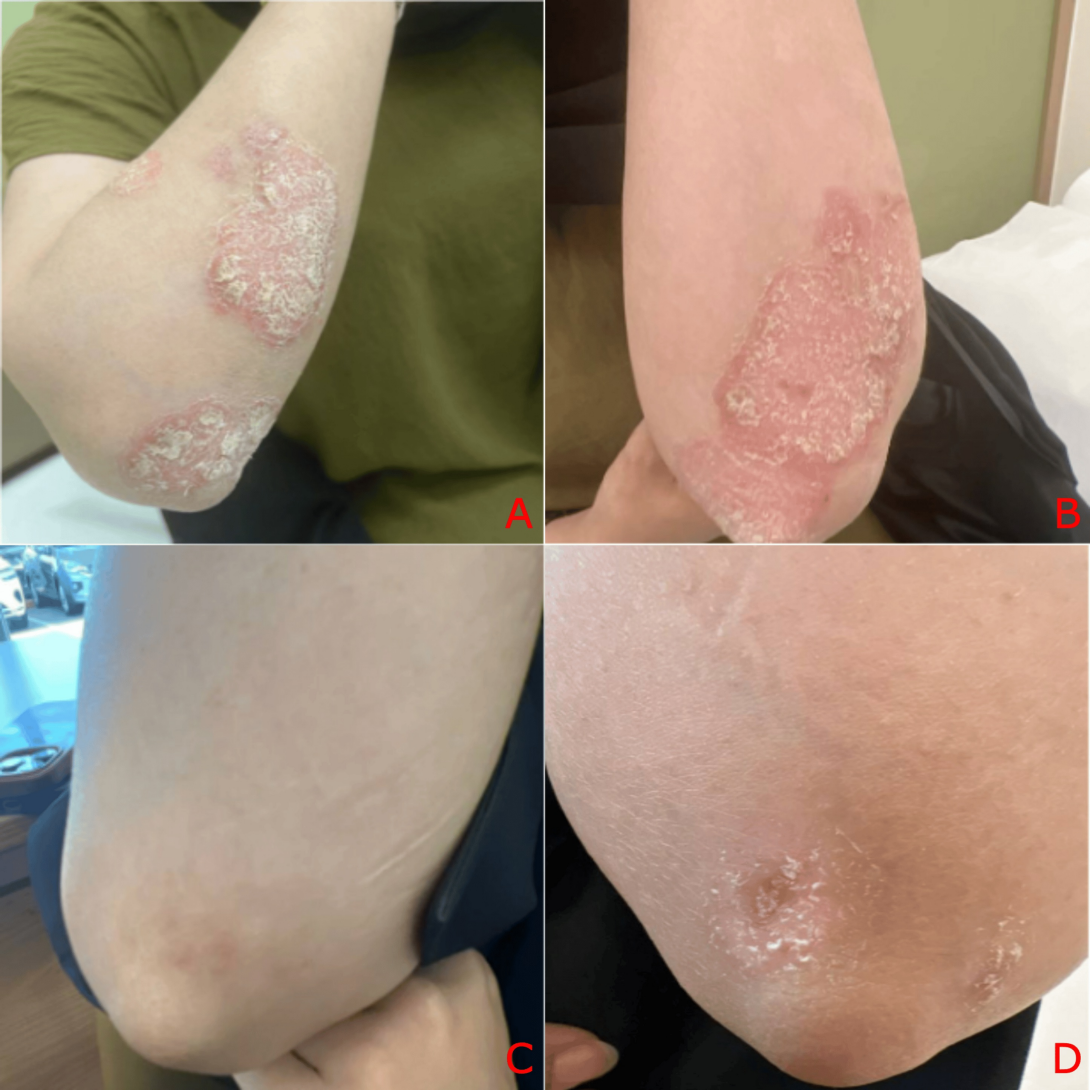
Psoriatic plaques on the right (A) and left (B) elbows prior to treatment with guselkumab and post 12 months of treatment (C,D)

In 2021, the patient developed a scaly rash on her scalp, shins, and elbows. A skin biopsy confirmed psoriasis (Figure 1). Initial treatment with topical therapy was unsuccessful. Secukinumab, an Interleukin-17 (IL-17) inhibitor, was trialled based on expert dermatological advice but did not result in satisfactory clinical improvement. Consequently, treatment was switched to ustekinumab, an IL-12 and IL-23 inhibitor, which unfortunately triggered a lupus flare and a herpes zoster infection. As a result, guselkumab, an IL-23 inhibitor, was initiated. After 17 months of treatment, the patient remains in remission for both lupus and psoriasis, with marked improvement in psoriasis, as demonstrated in Figure 2.

Case 2 

**Figure 3:**
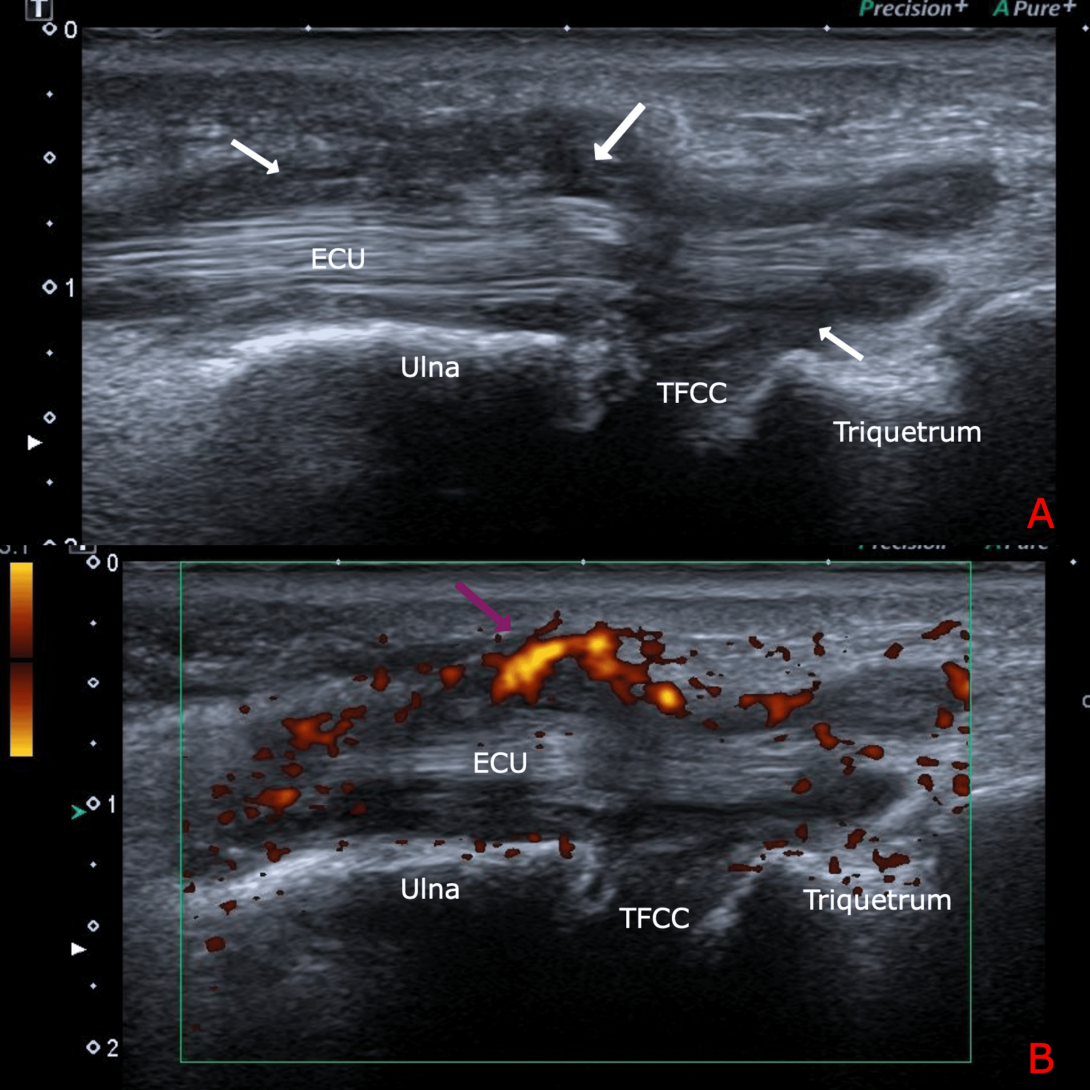
Long-axis ultrasound scan of the right wrist over the 6th extensor compartment The white arrows (A) point to severe tenosynovial hypertrophy of the 6th extensor compartment. The purple arrow (B) pointing to grade 3 power doppler signal indicating active severe ECU tenosynovitis. ECU: Extensor carpi ulnaris; TFCC: Triangular fibrocartilage complex

**Figure 4:**
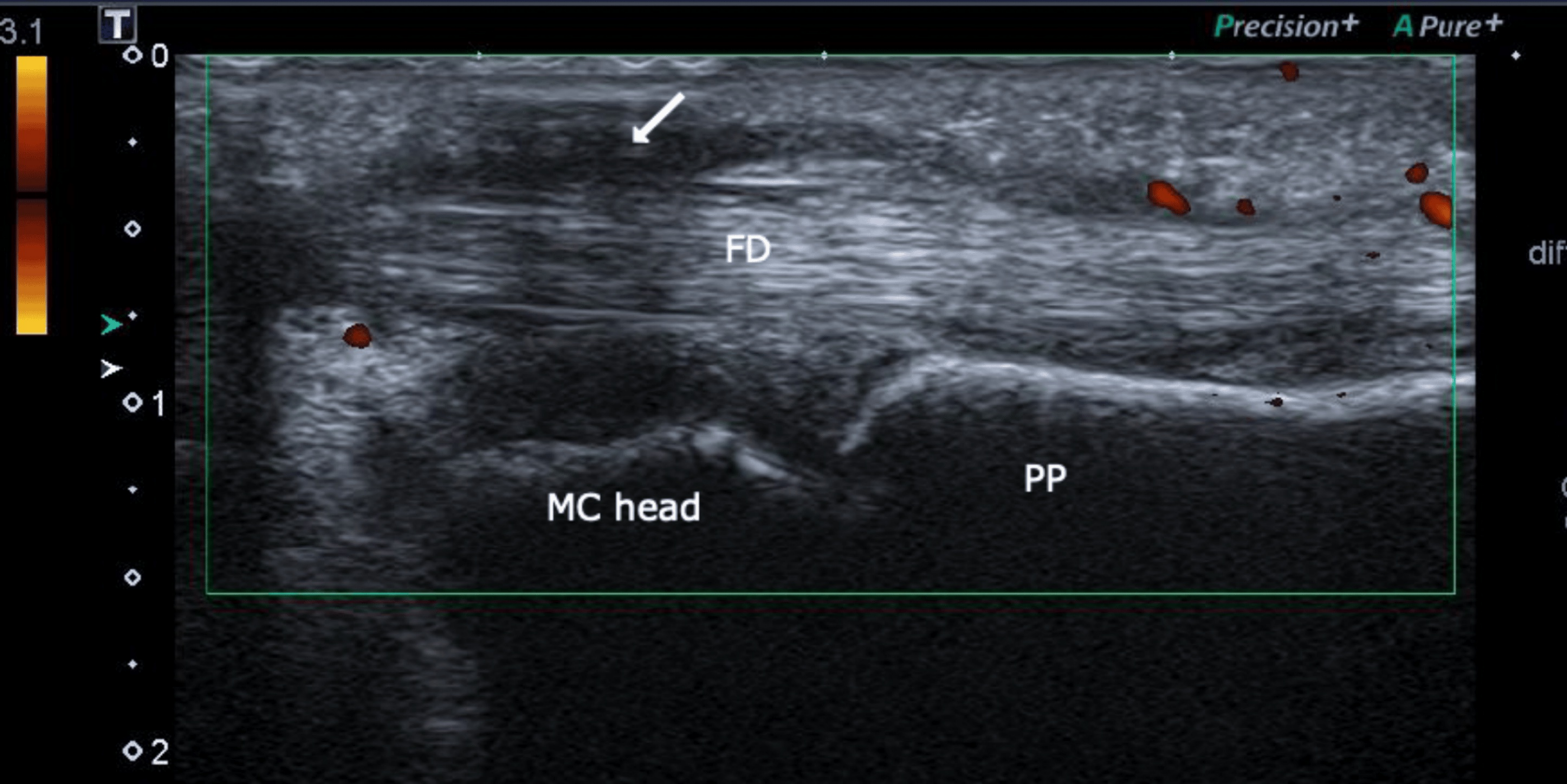
Long-axis ultrasound scan of the second flexor digitrorum of the right hand showing synovial hypertrophy (white arrow) FD: Flexor digitorum; MC: Metacarpal; PP: Proximal phalanx

**Figure 5:**
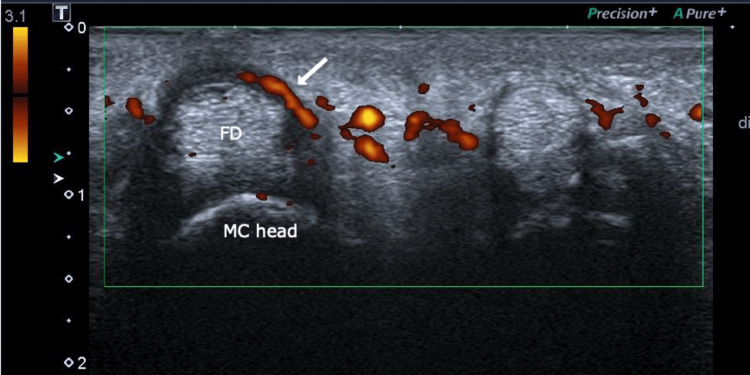
Transverse-view ultrasound scan of volar aspect of the second metacarpophalengeal joint of the right hand showing active tenosynovial power doppler signal (white arrow) FD: Flexor digitorum; MC: Metacarpal

**Figure 6:**
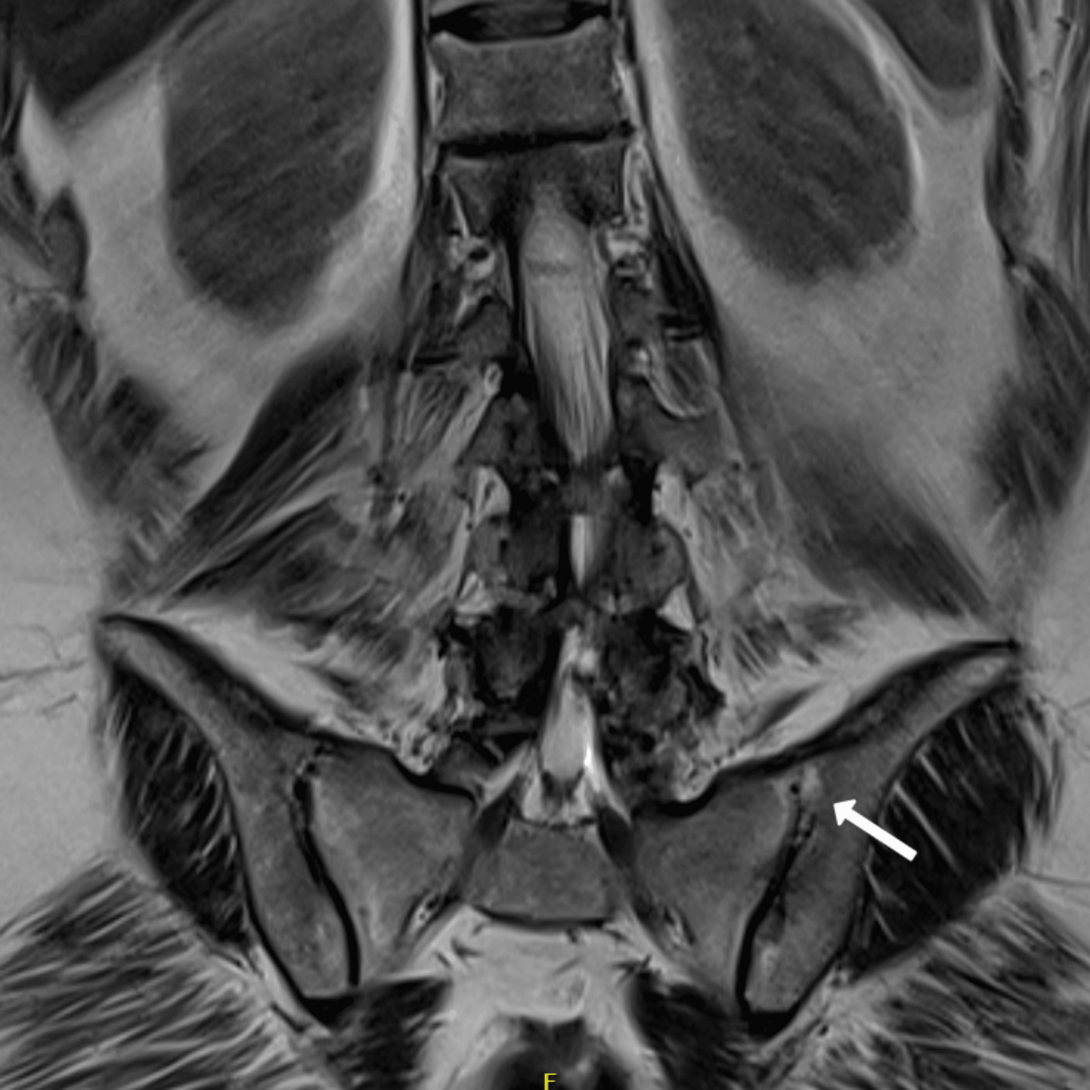
T2 fat-sensitive coronal section MRI of the sacro-iliac joints showing fatty metaplasia and ankylosis of the superficial margin of the left sacro-iliac joint (white arrow)

**Figure 7:**
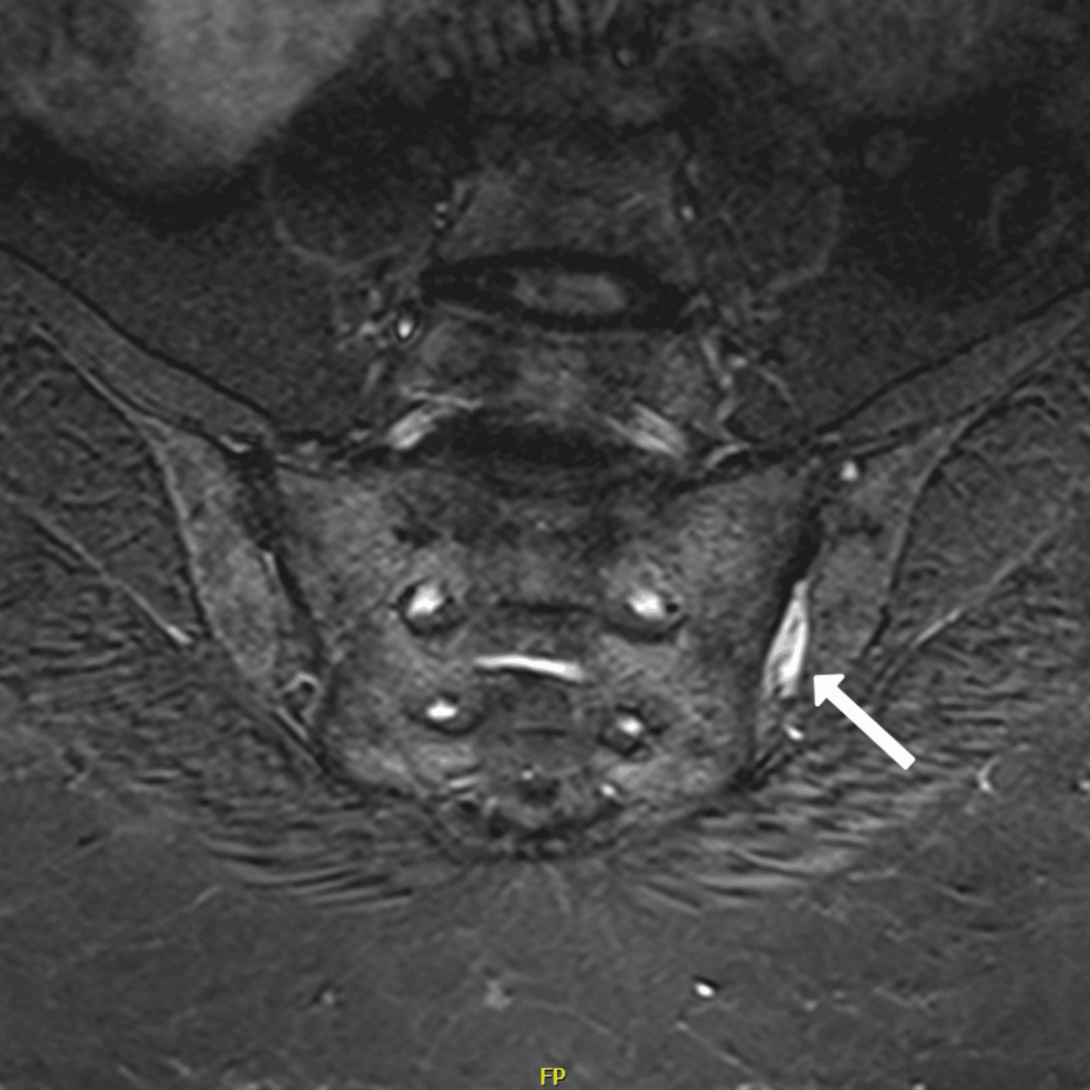
STIR-sequence MRI of the sacro-iliac joints showing left sided inflammatory changes (white arrow) STIR: Short tau inversion recovery

A 40-year-old female patient with a history of epilepsy, psoriatic arthritis (PsA) since 2007, and SLE diagnosed in 2024 presented with a complex autoimmune disease course. PsA was diagnosed clinically based on musculoskeletal findings, palmoplantar psoriasis, and the presence of dactylitis and tenosynovitis (Figures 3, 4, 5). Sacroilitis observed on MRI supported axial involvement consistent with PsA, as demonstrated in Figures 6, 7. Initially, she was prescribed methotrexate at a private clinic but was non-compliant due to financial constraints. In 2014, she presented with polyarticular joint pain, active psoriasis, and sacroiliitis; methotrexate, sulfasalazine, folic acid and a non-steroidal anti-inflammatory drug (NSAID) to cover the axial domain were re-initiated.

Over the years, she received multiple disease-modifying antirheumatic drugs (DMARDs) and biologics, including adalimumab, certolizumab, and IL-17 inhibitors, due to persistent disease activity. These biologic therapies were intermittently funded by charity organizations, leading to treatment interruptions when support lapsed. In February 2024, ixekizumab was introduced, resulting in significant improvement in her psoriasis. In May 2024, she was diagnosed with SLE, not based on musculoskeletal symptoms but on hematological abnormalities, positive ANA (which was previously negative in 2012) and anti-dsDNA antibodies, and low complement levels. Following this, she was started on methotrexate, prednisolone, and hydroxychloroquine at a low dose (200 mg daily) to minimize the risk of psoriasis exacerbation. She remains in remission for both PsA and SLE under regular follow-up.

Case 3 

A 30-year-old female with a history of SLE and Class II lupus nephritis, diagnosed in 2010, experienced multiple disease flares requiring various immunosuppressive therapies. In 2016, while receiving methotrexate and rituximab, she presented with a rash, epistaxis, and hemoptysis. Laboratory evaluation demonstrated positive SLE serologies with negative P-ANCA and C-ANCA, favoring a diagnosis of SLE over granulomatosis with polyangiitis (GPA). In the context of pregnancy planning, methotrexate and rituximab were discontinued and replaced with cyclosporine. Due to subsequent deterioration in renal function, therapy was transitioned to tacrolimus in 2017.

By 2020, her lupus nephritis had entered remission; however, she developed seizures, necessitating a switch from tacrolimus to azathioprine and initiation of levetiracetam. Following her pregnancy in 2020 and postpartum period in 2021, she experienced a severe flare of lupus nephritis and psoriasis. Management included intravenous methylprednisolone pulses (500 mg daily for three days), followed by oral prednisolone at 0.5 mg/kg/day for four weeks, with gradual tapering and discontinuation over a 24-week period. Although systemic corticosteroids are known to potentially exacerbate psoriasis, the decision to initiate high-dose corticosteroid therapy was based on a risk-benefit assessment prioritizing renal preservation over dermatologic concerns. Concurrent treatment included mycophenolate mofetil and re-initiation of tacrolimus.

**Figure 8:**
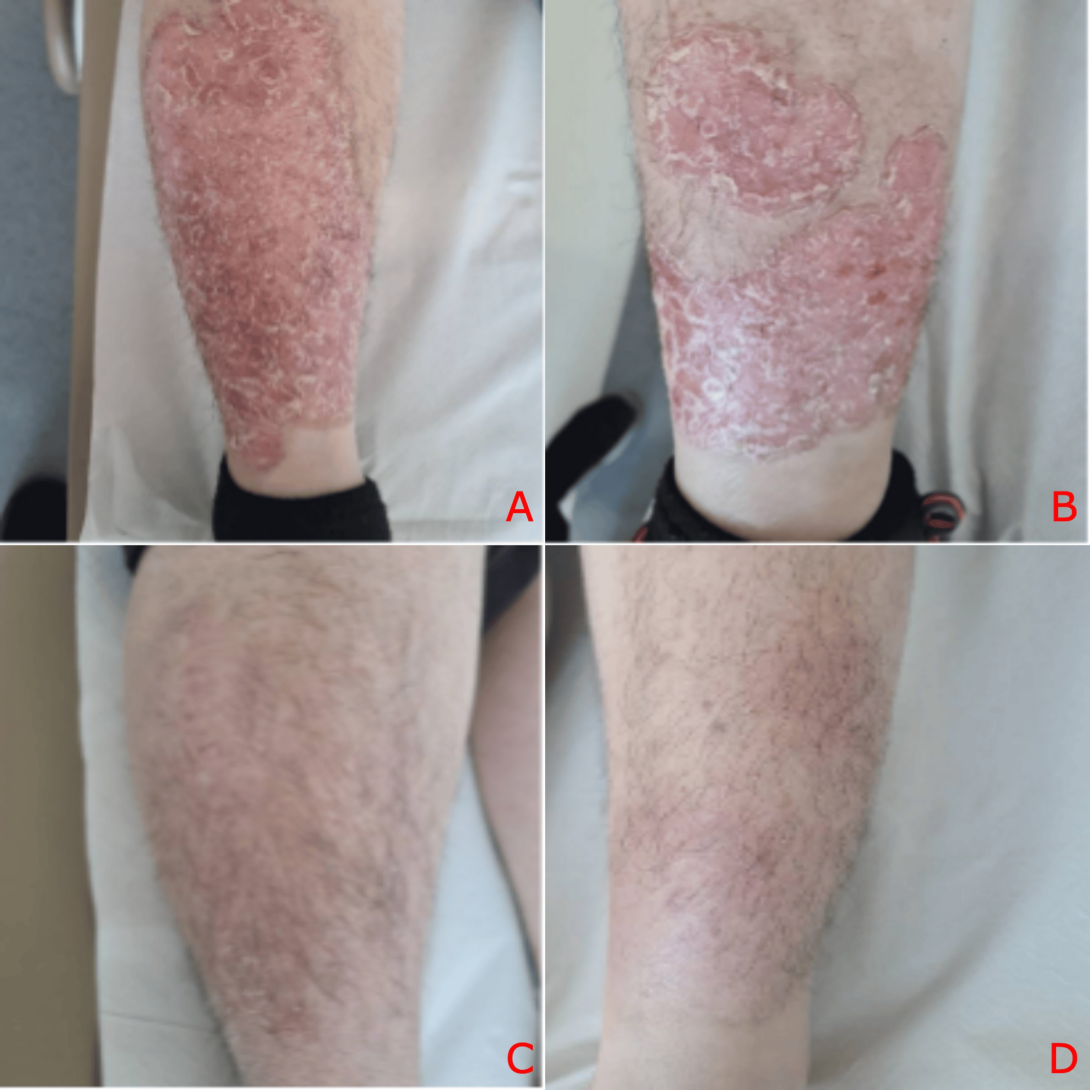
Psoriatic plaques on the right (A) and left (B) legs prior to treatment with Guselkumab and post three months of treatment (C,D)

In 2023, azathioprine was reintroduced in preparation for subsequent pregnancy, maintaining remission. However, in December 2024, she developed severe psoriasis, confirmed both clinically and histopathologically via skin biopsy reporting features of psoriasiform hyperplasia with focal parakeratosis, with diagnostic confirmation provided by dermatology specialists. Given the extent of her cutaneous disease, guselkumab, an IL-23 inhibitor, was initiated, resulting in marked clinical improvement, as demonstrated in Figure 8.

Case 4

A 39-year-old female with a history of SLE, diagnosed in April 2006, presented with a long-standing course characterized by cutaneous, musculoskeletal, hematological, and immunological involvement. Her initial symptoms included symmetric joint pain affecting the hands, elbows, knees, and feet; morning stiffness lasting for an hour; and a photosensitive malar rash. She also reported dryness of the eyes but denied oral ulcerations or features of Raynaud's phenomenon. Laboratory findings were notable for positive antinuclear antibodies, positive anti-Ro, and elevated anti-dsDNA. Complement levels (C3 and C4) were low. She was diagnosed with SLE with secondary SS, with no major organ involvement, and initiated on a regimen of prednisolone (15 mg daily) and hydroxychloroquine (200 mg daily).

Over the years, the patient experienced intermittent flares, often triggered by physical or emotional stress, resulting in musculoskeletal involvement and skin rashes. In 2017, she was started on methotrexate due to persistent arthritis, but this was later replaced with biologic therapy (belimumab) in 2022 after a moderately active lupus flare. Despite some improvements, belimumab was discontinued in January 2023 due to the emergence of suspicious breast dysplastic lesions.

**Figure 9:**
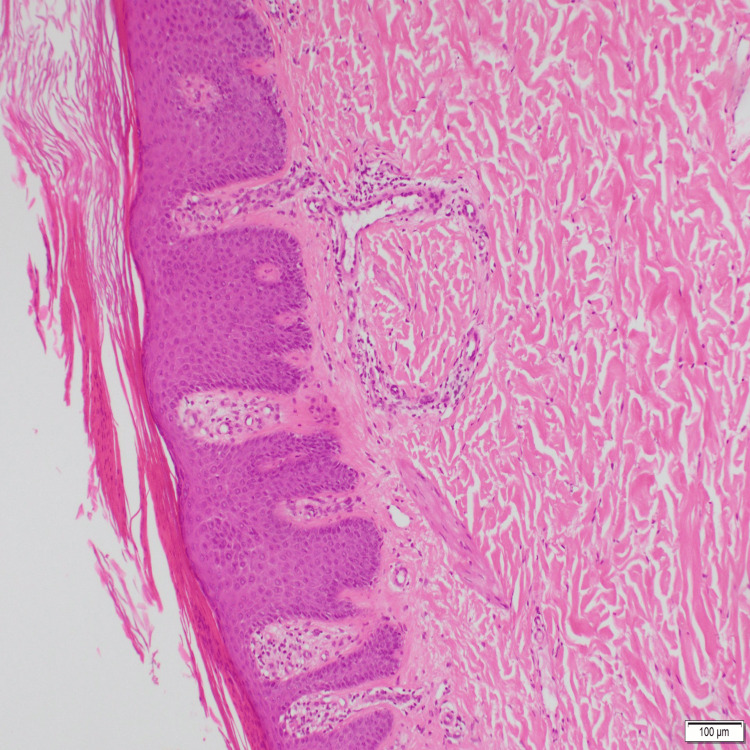
Skin biopsy showing features of psoriasiform mildly spongiotic dermatitis (low power 10x) Microscopic description reveals the presence of focal follicular plugging, focal hypergranulosis, alternating layers of orthokeratosis and parakeratosis without neutrophils.

In April 2024, she developed a new cutaneous flare, with erythematous, scaly plaques over the extensor surfaces of her legs, clinically suggestive of psoriasis. A skin punch biopsy confirmed psoriasiform dermatitis, with histopathological features most consistent with psoriasiform hyperplasia (Figure 9). The patient was referred to the dermatology department, where she was started on ixekizumab, an IL-17 inhibitor, for moderate to severe psoriasis. By February 2025, the patient demonstrated a favorable response to therapy, with significant resolution of the psoriasiform rash and no new signs of lupus activity.

Table 1 provides a summary of the clinical data recorded from the participants.

**Table 1:** Table showing the antibodies and the biochemical profile for all of the patients upon diagnosis with SLE ANA: Antinuclear antibody; Anti-dsDNA: Anti-double stranded DNA; Anti-RNP: Anti-ribonucleoprotein particle; Anti-SM: Anti-Smith; SS-A: Sjögren's syndrome-related antigen A; SS-B: Sjögren's syndrome-related antigen B; CRP: C reactive protein; ESR: Erythrocyte sedimentation rate

	Reference Range	Case 1	Case 2	Case 3	Case 4
ANA	< 1/100	1/1,000	1/100	1/320	1/1,000
Anti-dsDNA	< 100 IU/ml	666 IU/ml	483.65 IU/ml	232 IU/ml	380 IU/ml
C3	0.90-1.80 g/L	0.53 g/L	0.46 g/L	0.92 g/L	0.64 g/L
C4	0.10-0.40 g/L	0.03 g/L	0.04 g/L	0.07 g/L	< 0.1 g/L
Ribosomal P-proteins	Negative (< 1.0)	Negative	Negative	Negative	Not available
Anti-RNP	Negative (< 1.0)	Negative	Negative	Negative	Not available
Anti-SM	Negative (< 1.0)	Negative	Negative	Negative	Not available
SS-A (RO)	Negative (< 1.0)	Positive	Positive	Negative	Positive
SS-B (LA)	Negative (< 1.0)	Negative	Negative	Negative	Not available
SCL-70	Negative (< 1.0)	Negative	Negative	Negative	Not available
JO-1	Negative (< 1.0)	Negative	Negative	Negative	Not available
Centromeres	Negative (< 1.0)	Negative	Negative	Negative	Not available
CRP	< 10 mg/ L	< 10 mg/L	31 mg/L	< 10 mg/L	< 10 mg/L
ESR	0-12 mm/1hr	18 mm/1hr	122 mm/1hr	85 mm/1hr	55 mm/1hr

## Discussion

SLE is a chronic multisystem autoimmune disease that primarily affects females at the child reproductive age [4]. The prevalence of SLE varies from one region to another based on sociodemographic factors with an estimated global prevalence of 43.7 per 100,000 persons [5]. Psoriasis is a chronic inflammatory skin condition that affects both adults and children with an estimated worldwide prevalence of 0.9 to 8.5 percent in adults and 0 to 2.1 percent in children. Psoriasis affects both sexes equally and peak ages for the onset of psoriasis are between the ages of 30 to 39 years and 50 to 69 years [6].

SLE is known to coexist with other connective tissue diseases in an entity called overlap syndromes. These include overlap with RA, SS, SSc, DM, and PM. Recognizing overlap syndromes is important for understanding disease prognosis and optimizing management. The overlap between SLE and psoriasis is relatively rare and has been reported in case reports and few retrospective case studies. According to one retrospective study in 1996, 0.23% of 9420 patients with psoriasis were found to have SLE [7]. Another retrospective study done in 2016 showed that psoriasis prevalence was 5.1% among 445 patients with SLE [8].

The overlap of SLE with psoriasis poses a significant diagnostic and therapeutic challenges due to the overlapping clinical, immunological, and therapeutic considerations. For instance, both conditions can cause erythematous plaques, scaling, and photosensitivity, making it difficult to distinguish between lupus erythematosus cutaneous manifestations and psoriatic skin lesions. Arthropathy can also pose a diagnostic challenge as both conditions can present with seronegative arthralgia and PsA may precede any skin manifestations. However, PsA is typically oligo-articular, asymmetric and erosive, while SLE arthritis is usually non-erosive and symmetric.

In terms of management, hydroxychloroquine which is one of the key medications in the management of SLE can cause flares of psoriasis [9]. Moreover, guidelines typically discourage the use of systemic corticosteroids in patients with psoriasis due to concerns about potential flare-ups after steroid use. However, this recommendation is being increasingly questioned. A 2019 systematic review that included 11 retrospective and prospective cohort studies found that 10 observational and interventional studies did not demonstrate an increased risk of psoriasis flares with corticosteroid use. Consequently, the review concluded that the risk is low and that the evidence supporting this guideline is weak [10]. Moreover, phototherapy, which is commonly used in the treatment of psoriasis skin lesions, can lead to worsening photosensitive skin rash and systemic symptoms such as fatigue and arthralgia in patients with SLE [11]. Tumor necrosis factor-alpha (TNF-A) antagonist who has shown great success in controlling both psoriasis and PsA, has been reported to precipitate existing SLE. It can also cause an interesting type of drug induced lupus where anti-histone antibodies are less commonly found compared to patients with drug induced lupus secondary to other medications [12].

The pathophysiology of SLE involves dysregulation in both innate and adaptive immune responses, characterized by impaired apoptotic cell clearance and dysfunctions in B- and T-cell activity [4]. In the contrary, psoriasis is a chronic auto immune inflammatory skin disease that is predominantly a T-cell-mediated disorder driven by the IL-23/Th17/IL-17 pathway. This pathway plays a central role in driving a self-sustaining inflammatory cascade, leading to dysregulated keratinocyte proliferation and differentiation, which contributes to the development of characteristic psoriatic lesions. Elevated levels of IL-23 were found to positively correlate with disease activity, with increasing levels found in skin lesions during active psoriasis during active psoriasis and declining following successful treatment [13]. However, circulating plasma IL-23 levels do not exhibit a significant association with disease severity as measured by the Psoriasis Area and Severity Index (PASI) or affected body surface area (BSA) [14]. 

Studies have demonstrated that the IL-17/IL-23/TNF-A axis plays a crucial role in mediating inflammatory responses and contributing to end-organ damage in SLE. IL-17, in particular, promotes B-lymphocyte differentiation, and elevated levels of both IL-17 and IL-23 have been identified in renal biopsy specimens from patients with lupus nephritis compared to healthy controls. Supporting these observations, Wong et al. reported significantly higher plasma IL-17 concentrations and an increased frequency of Th17 cells in SLE patients relative to healthy individuals [15]. Additionally, Tang et al. found a positive correlation between circulating IL-17 levels and disease severity, as assessed by the SLE Disease Activity Index (SLEDAI) score, further underscoring the cytokine’s role in SLE pathogenesis [16]. Importantly, the involvement of the IL-17/IL-23/TNF-A axis is not only limited to SLE. This pathway is also central to the pathogenesis of psoriasis, a disease thought to be mediated by Th17 and Th22 cells. Therefore, dysregulation of this shared immunological axis may contribute to psoriasis flares in patients with coexisting SLE. However, to our knowledge, no studies have specifically investigated plasma IL-23 levels in patients with overlapping SLE and psoriasis compared to control groups, as the available studies have focused solely on SLE without concurrent psoriasis.

TNF-A also plays a major role in the pathogenesis of both SLE and psoriasis. It also has a concentration dependent action as it is found to have pro-apoptotic and pro-inflammatory actions at higher concentrations and anti-apoptotic and anti-inflammatory action at lower concentrations. In psoriasis, higher levels of TNF-A stimulate the proliferation and differentiation of T and B lymphocytes and increases the number of dendritic cells which consequently upregulate the production of a range of pro-inflammatory cytokines and ILs that contribute to the inflammatory process. High TNF-A levels also play a role in the pathogenesis of SLE as it has a pro-apoptotic effect, causing increased auto antigen exposure that leads in increased auto antibodies and ultimately development of an auto immune process [17]. TNF inhibitors should theoretically be an excellent candidate in the treatment of SLE-psoriasis overlap, data has shown that although it has an excellent response in treatment of psoriasis, there are reports of worsening SLE cutaneous and systemic manifestations. In addition to possibility of TNF-A drug induced lupus in patients with no prior diagnosis of lupus erythematosus [12].

The presence of anti-Ro antibodies has also been found to be associated with SLE and psoriasis overlap. These antibodies exhibit a positive correlation with photosensitivity, as ultraviolet (UV) radiation upregulates Ro antigen expression on keratinocyte surfaces. Elevated serum anti-Ro antibody levels can subsequently trigger cytotoxicity and an antibody-mediated immune response, potentially leading to basal epidermal layer damage and cutaneous lesions, particularly in sun-exposed areas. Anti-Ro antibodies are most frequently detected (70-90%) in subacute cutaneous lupus erythematosus (SCLE) patients. This variant of lupus is distinguished by a higher prevalence of psoriasis-like skin lesions and more pronounced photosensitivity compared to other lupus forms [18].

The literature on SLE-psoriasis overlap remains limited, as the condition is rare, with only a few retrospective case studies and case reports available. A study conducted by Bonilla et al. demonstrated that the prevalence of PsA in patients with SLE-psoriasis overlap was 83.1%, suggesting that these patients are at a significantly higher risk of developing PsA compared to those with psoriasis alone [8]. This association is complex, as both SLE and psoriasis can independently lead to arthritis, making it challenging to delineate their contributions to joint pathology. Additionally, photosensitivity and cutaneous manifestations characteristic of SLE have been observed to be more prevalent in patients with SLE-psoriasis overlap compared to those with SLE alone [8]. The presence of anti-Ro antibodies has been strongly associated with photosensitivity in SLE, and a small study involving four patients with SLE-psoriasis overlap reported a higher prevalence of anti-Ro antibodies in these individuals compared to SLE patients without psoriasis [19]. 

These findings highlight a potential avenue for future research, particularly in elucidating the relationship between anti-Ro antibodies and photosensitivity, as well as their significance in the context of SLE-psoriasis overlap. The use of antimalarial agents, which are known to exacerbate psoriasis, has also been reported to induce psoriasis in patients with no prior history of the disease. Therefore, obtaining a detailed patient history, including prior use of antimalarial medications, is crucial, and clinicians should remain vigilant regarding this potential association.

In our study, we reported four patients with a prolonged and complicated disease course, characterized by multiple flares and remissions necessitating frequent modifications to their treatment regimens. Notably, the introduction of IL-17 and IL-23 inhibitors resulted in significant improvement of both lupus and psoriasis manifestations, with no subsequent complications. These findings underscore the potential utility of IL-17/IL-23 inhibitors in managing SLE-psoriasis overlap, particularly in patients with refractory disease.

Emerging evidence suggests that tyrosine kinase 2 (TYK2) plays a critical role in the pathophysiology of both SLE and psoriasis. TYK inhibitors have been approved for the treatment of moderate-to-severe psoriasis in several countries, demonstrating promising efficacy. Furthermore, controlled clinical trials assessing their use in SLE have reported significant improvement in both systemic and cutaneous manifestations [20]. However, additional research is required to establish their efficacy and safety profile in patients with SLE-psoriasis overlap.

Our case series is subject to few limitations. Some patients were initially diagnosed and treated at different medical centers, resulting in incomplete baseline investigations and antibody profiles for data collection. Additionally, several patients were managed across multiple rheumatology and dermatology clinics, which may have impacted treatment adherence and influenced disease progression. Financial constraints further posed a significant challenge, as some patients were unable to afford biologic therapies, leading to delays in initiation and interruptions in treatment. These factors likely contributed to disease activity and underscore the importance of addressing socioeconomic barriers in optimizing patient outcomes.

## Conclusions

The overlap of SLE and psoriasis presents unique diagnostic and therapeutic challenges due to shared clinical features and distinct immunopathogenic pathways. Our case series highlights the complexity of managing these patients, especially in the presence of comorbidities and limited access to advanced therapies. IL-17 and IL-23 inhibitors demonstrated promising results in controlling disease manifestations without significant complications. Further research is warranted to deepen our understanding of the immunological interplay between SLE and psoriasis and to optimize treatment strategies for this rare but clinically significant overlap syndrome.
